# Spontaneous splenic pseudocyst: Case report of a rare entity

**DOI:** 10.1002/ccr3.6964

**Published:** 2023-02-23

**Authors:** Govinda Prasad Tiwari, Ramchandra Poudel, Sabin Nepal, Sushil Dhakal

**Affiliations:** ^1^ Department of General Surgery Navajeevan Hospital Pvt Ltd Kailali Nepal; ^2^ Department of Internal Medicine Nova Hospital Pvt Ltd Kailali Nepal; ^3^ Department of Radiodiagnosis, Imaging and Intervention Nisarga Hospital and Research Centre Kailali Nepal; ^4^ Department of Pathology Mayametro Hospital Pvt Ltd Kailali Nepal

**Keywords:** pseudocyst, spleen, splenic cysts, surgery

## Abstract

Splenic cysts are classified on the basis of epithelial lining, either primary or secondary. Primary cysts are further divided as parasitic and nonparasitic. The secondary cysts are usually post traumatic or after a splenic extension of pancreatic pseudocyst. However, not all pseudocysts are associated with trauma. Mostly, they are asymptomatic (30%–60%) and usually grow in size to cause compressive symptoms. Splenic pseudocysts should be differentiated with other malignant and nonmalignant pathology, specifically hydatid cysts, in order to manage them correctly. The walls of pseudocysts may be degenerative or calcified, which may resemble hydatid cysts. Here, we present a case of a non‐traumatic splenic cyst masquerading as a hydatid cyst preoperatively. The patient was taken up for surgery and intraoperatively noted to be a hemorrhagic cyst with a non‐splenic cyst wall. We decided to preserve the spleen with marsupialisation of cyst and omentoplasty. On histopathology, the diagnosis of a pseudocyst of spleen was made in view of absent epithelial lining. We would like to report this case because of the diagnostic dilemma, its clinical rarity and, even more, in the absence of any history of trauma.

## Graphical abstract

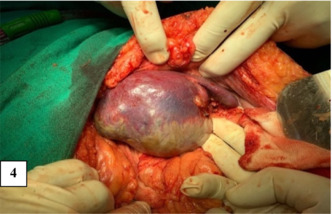

## 1 INTRODUCTION

Splenic cysts are very rare, occurring in around 0.07% of the world population. Only 800 cases are reported in the world literature, and one of the rarest forms is the pseudocyst of spleen.^1^ Owing to their vast heterogeneity, they are classified as primary or true (those with epithelial lining) and secondary or false/pseudocyst (without epithelial lining). Pseudocysts are hemorrhagic (post traumatic) or serous. Parasitic cysts are the most common of all and are secondary to Echinococcus granulosus.^2^ These cysts are mostly asymptomatic and are diagnosed on incidental scans done for other causes.^3^, ^4^ Secondary cysts are primarily due to abdominal trauma and, to a lesser extent, due to tuberculosis, malaria, or infectious mononucleosis.^5^ Small cysts less than 5 cm are usually asymptomatic and larger cysts more than 8 cm are likely to be symptomatic. The symptoms are due to gradual compression of the surrounding structures and expansion of the size of the cyst itself. They may present with left upper quadrant or epigastric pain, early satiety due to stomach compression, fever due to abscess or hypersplenic sequestration (anemia or thrombocytopenia).^6^ It is important to distinguish the pseudocyst of spleen from other benign/malignant splenic cyst specially from hydatid cyst as they have different management modalities. Surgery is the gold standard for larger cysts, which can be in the form of splenectomy or splenic preservation (partial splenectomy, marsupialization, or fenestration).^1^, ^7^, ^8^, ^9^

## 2 CASE PRESENTATION

**FIGURE 1:**
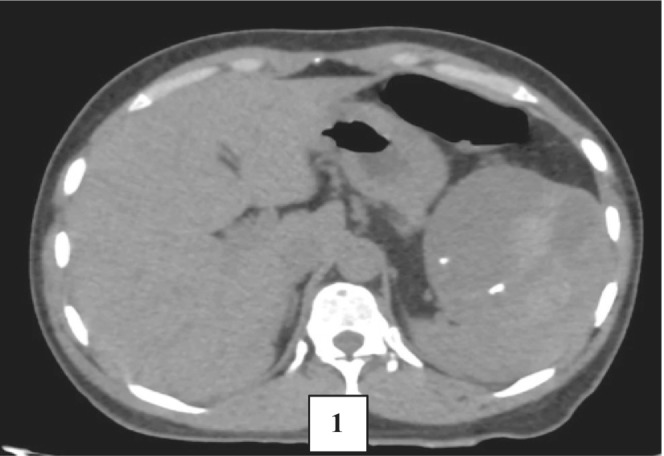
Plain axial CT of the cyst showing areas of relative hyperdensity and speckled calcification within.

**FIGURE 2:**
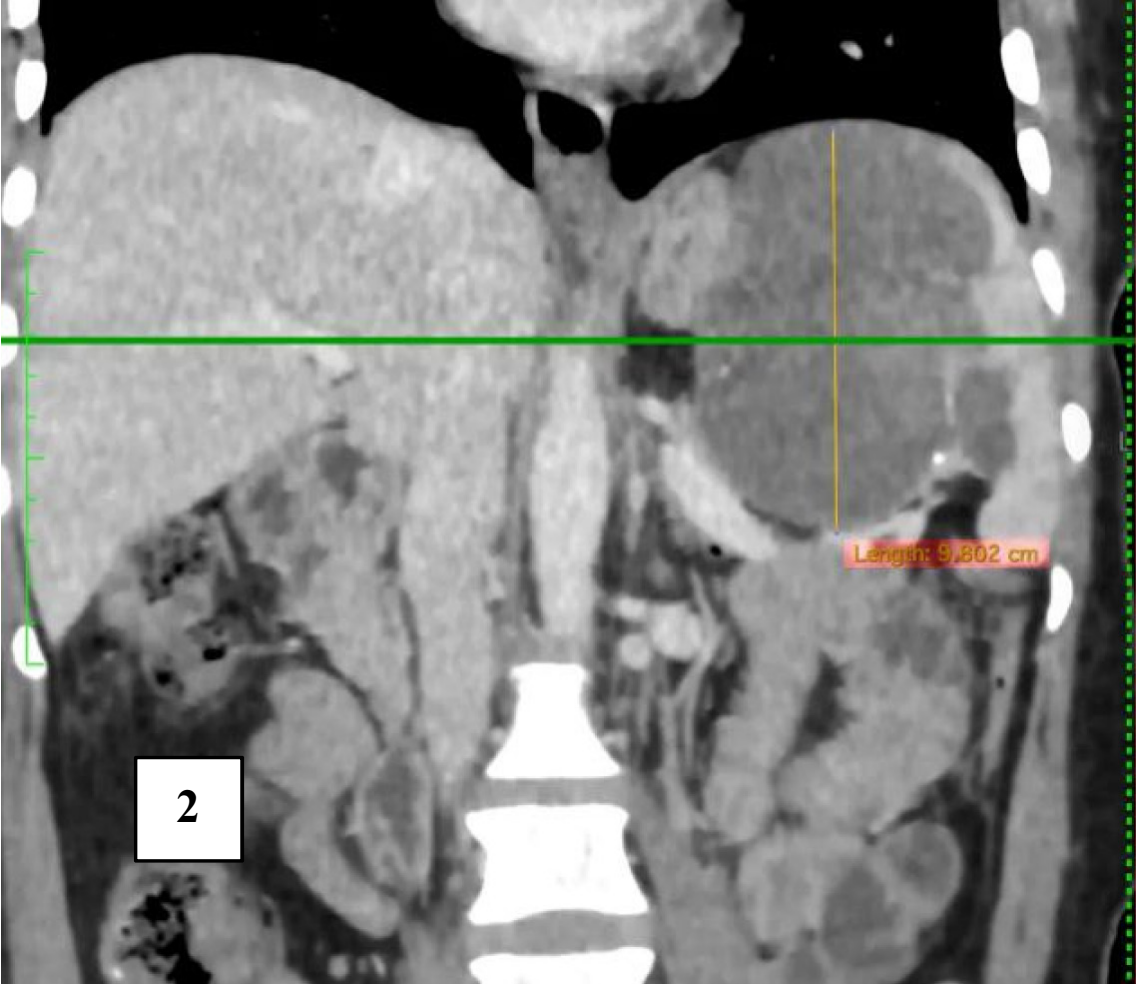
Coronal View: in the venous phase shows the cystic lesion occupying the hilar region of the spleen, extending towards the superior pole. Note is made on splenic vein inferiorly.

**FIGURE 3:**
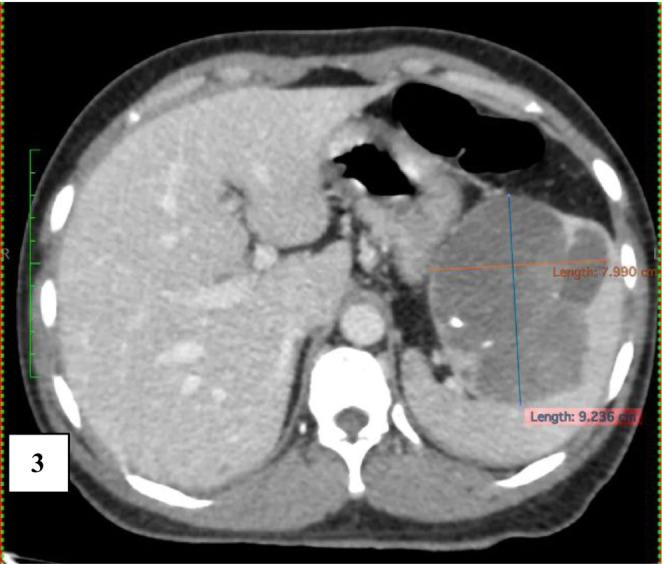
Coronal View: In the venous phase shows the cystic lesion occupying the hilar region of the spleen, extending towards the superior pole. The lesion is abutting splenic vein inferiorly and cardia anteromedially with intact fat planes in between.

A 34‐year‐old lady under evaluation for recurrent vaginal discharge was referred by the department of Gynecology for a splenic cyst, likely a hydatid cyst detected on ultrasonography, as an incidental and sole finding. The patient also complains of left upper abdominal pain, early satiety, and heaviness in the epigastric region. There was no history of fever, cough, jaundice, and symptoms of upper GI bleeding. There was no history of any abdominal trauma, contact sports previously, or previous surgery. She was a non‐smoker and non‐alcoholic and was under the empirical treatment for vaginal discharge. On examination, she had no pallor and was non‐icteric. The abdomen was soft and non‐tender with no palpable abdominal mass. Contrast enhanced computed tomography of the abdomen and pelvis showed a well‐defined, round large cystic lesion measuring 7.8 × 8.8 × 9.6 cm (AP × TR × CC) seen in the hilar region of the spleen extending into the upper pole. The cystic lesion showed multiple thin walled septations and calcifications within. There were subtle hyperdense contents in the dependent part of the individual loculi within the cyst. Medially, the lesion abutted the pancreatic tail and cardia of the stomach with a maintained fat planes in between. The lesion appeared to compress the stomach cardia supero‐medially and compress the splenic vein inferiorly. There was no wall calcification, no surrounding collection, or fat stranding. Following the contrast administration, the cystic lesion, including the hyperdense content, thin septa, and cyst wall, did not show any significant enhancement (Figures 1, 2, 3). The differential diagnosis was hydatid cyst (Gharbi III), cystic neoplasm of spleen.

**FIGURE 4:**
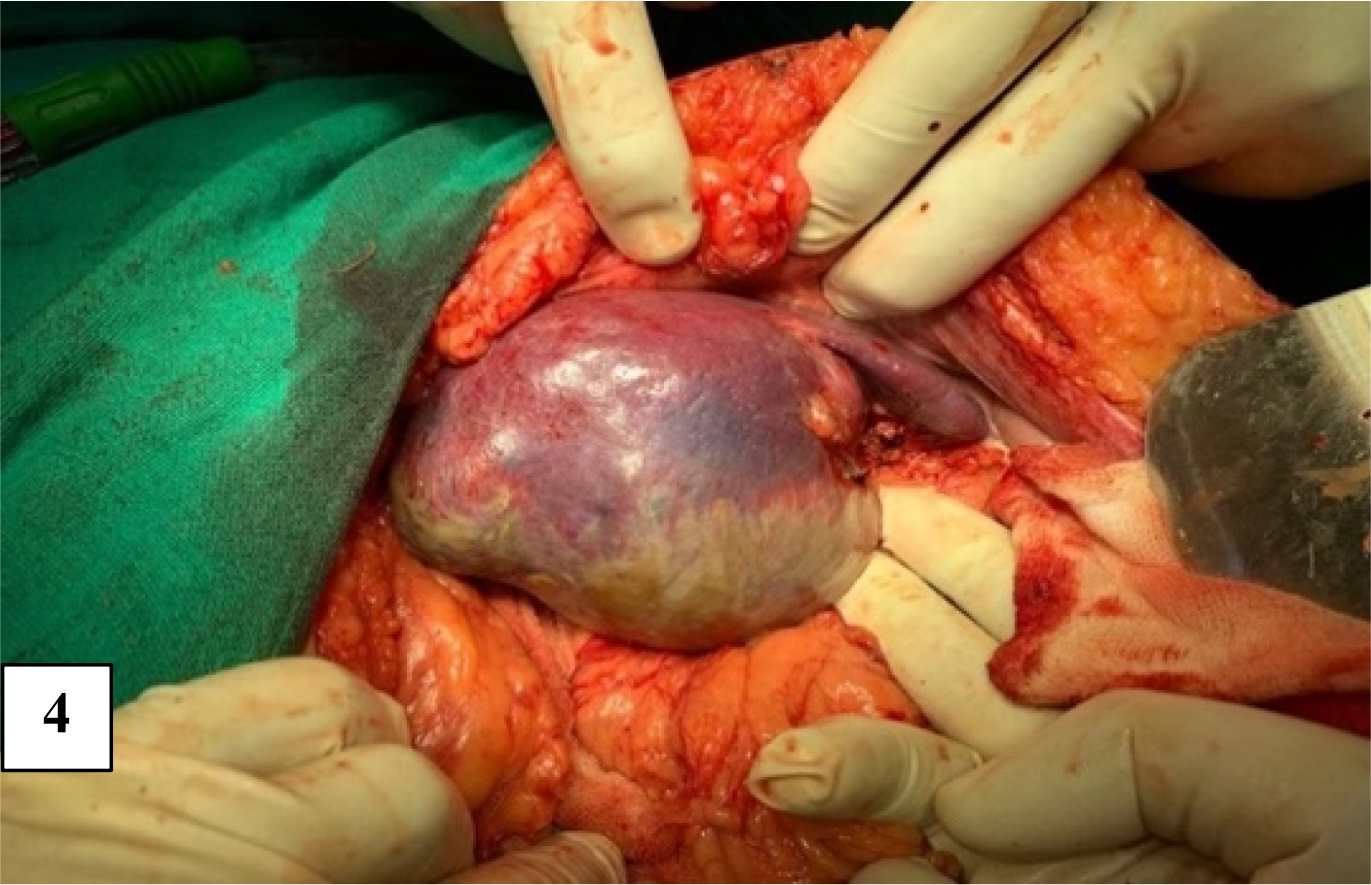
Intraoperative image of the cystic lesion at the upper pole of the spleen.

Hematological and biochemical parameters were under normal limits. Echinococcus IgG ELISA was negative. Preoperative preparation included Nil per oral protocol for 8 h, preoperative prophylactic iv. antibiotics one hour prior to incision and underwent an elective exploratory laparotomy under general anesthesia. Intraoperative findings noted was a large 10 × 8 cm splenic cyst with soft to firm walls, originating from the upper pole of the spleen. The outer wall of the cyst was separate from the splenic tissue, and the splenic vein did not pass through the cystic wall. The cyst was firmly adherent to the medial walls of the spleen (Figure 4).

**FIGURE 5:**
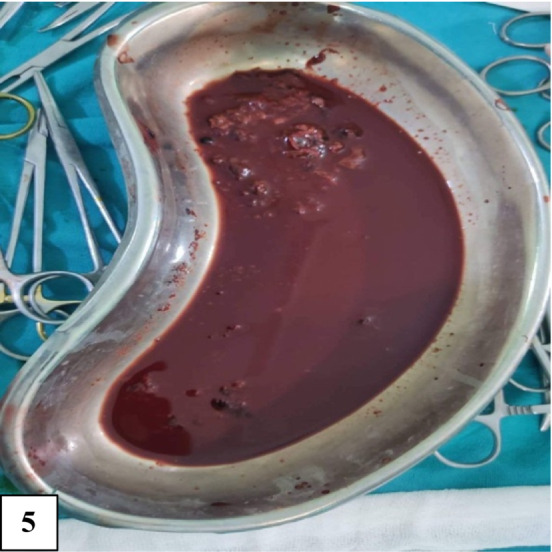
Image showing the hemorrhagic content of the cyst.

**FIGURE 6:**
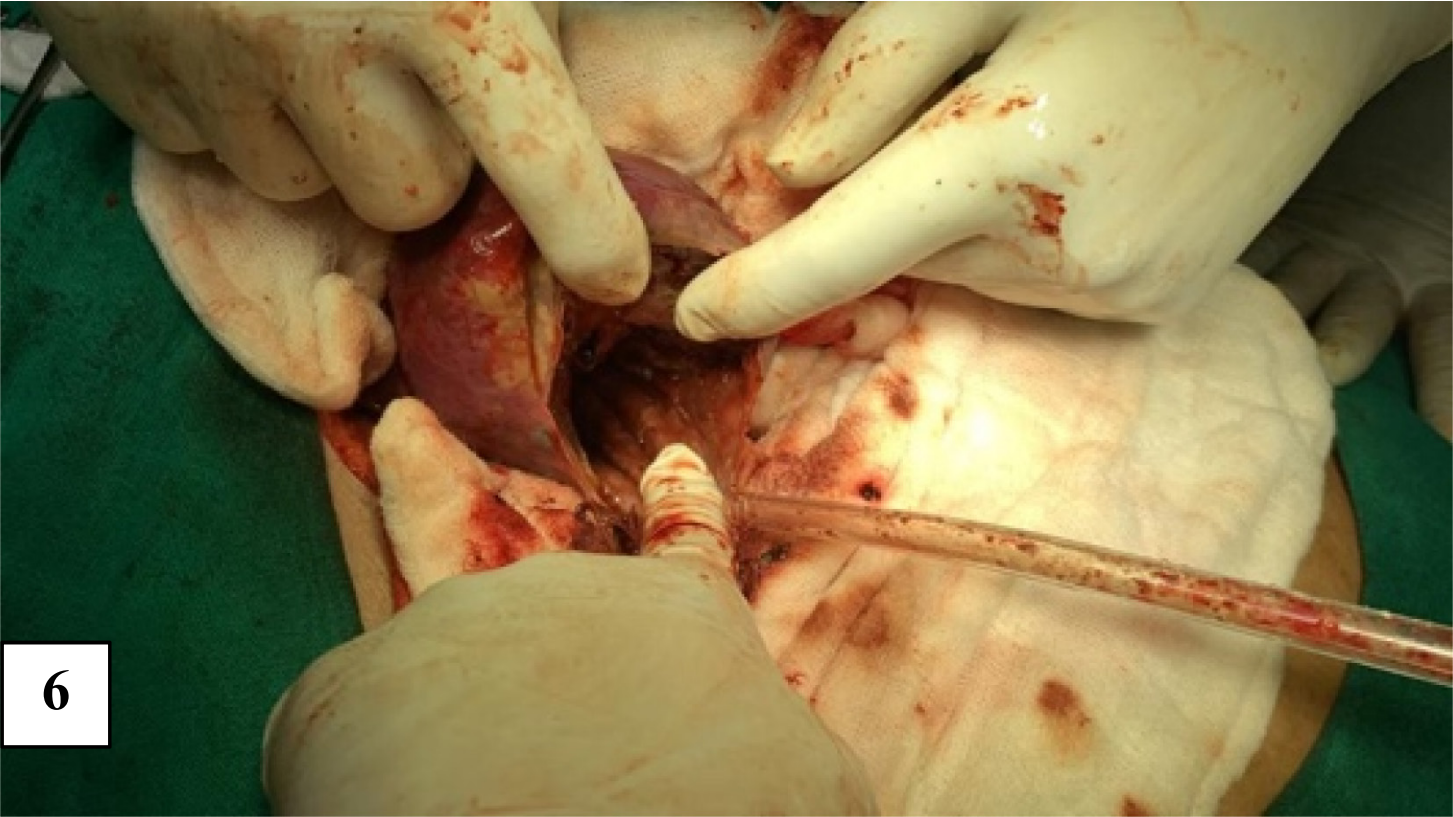
Image showing the marsupialisation of the cyst.

**FIGURE 7:**
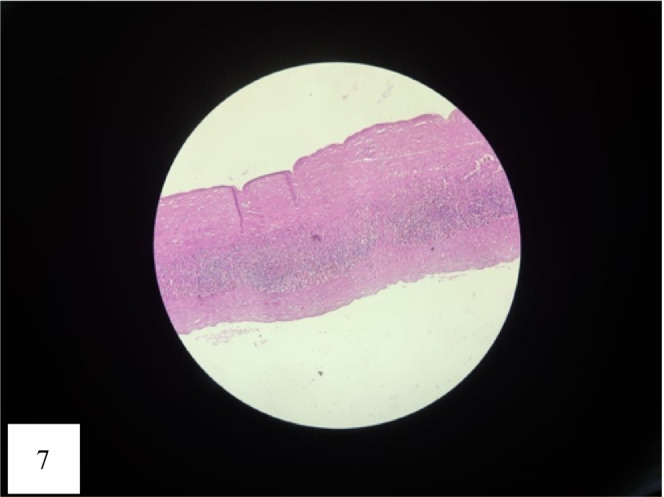
Microphotograph revealing cyst with lining epithelium and a fibrotic wall (H &E, × 20).

**FIGURE 8:**
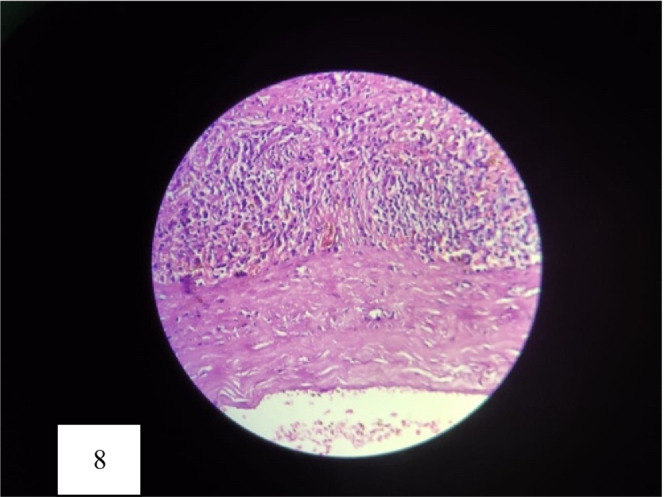
Microphotograph shows cyst devoid of any lining epithelial cells. Wall is fibrotic and infiltrated by chronic inflammatory cells and hemosiderin laden macrophages (H& E, × 400 times).

**FIGURE 9:**
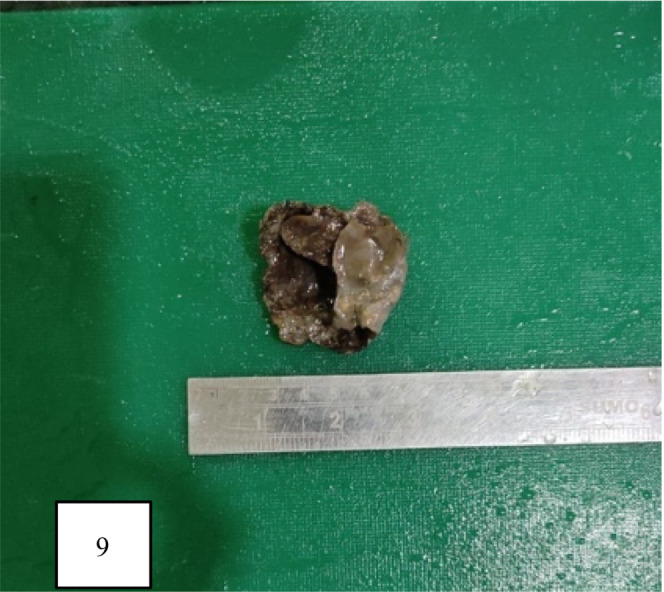
Gross image of cyst. Inner surface is irregular with areas of blackish discoloration.

Cyst was first aspirated to see the content that was altered/old blood without clots (Figure 5). Marsupialisation of the cyst with removal of the contents was done (Figure 6). The remaining part of the cyst was obliterated using the omental pedicle. The postoperative stay was uneventful, and the patient was discharged after 2 days of hospital stay. After 1 month of follow‐up, the patient was doing well and no fresh complaints were noted. The clinical outcome is similar to the outcome expected by the patient. She is kept under interval follow‐up with ultrasonography to see the recurrence. Histopathological examination revealed a pseudocyst without an epithelial lining and a fibrotic wall (Figures 7 and 8). The inner wall of the cyst was irregular with blackish discoloration (Figure 9).

## 3 DISCUSSION

Splenic cysts are a very rare entity, and only around 800 cases have been reported as per the literature. The first classification of splenic cysts was done as true or false cysts. True cysts were either parasitic or non‐parasitic. Almost three fourths of all the non‐parasitic cysts are comprised by pseudocysts. They are mostly due to trauma, infarction, or infection.^8^ Splenic pseudocysts are more common than true cysts, and 75% are due to trauma.^1^ That means only 25% of those pseudocysts are non‐traumatic,^10^ as in our case. Clinically, most of the pseudocysts are asymptomatic and rarely complicate.^10^, ^11^ Cowles et al.^12^ found that out of 191 cases of splenic cysts, only 5.2% were complicated with infection and rupture. Symptoms are proportionate with the size and location of the cyst.^10^, ^13^ Pressure symptoms constitute early satiety, vomiting, flatulence, pleuritic chest pain, dyspnea or persistent cough, left shoulder pain, or may present as a palpable mass.^1^, ^10^ Pseudocysts may rise to a significantly larger size and may contain as much as three liters of turbid fluid and are thought to be the final stage of organization of intrasplenic hematoma. The induction of chronic inflammatory process forms a fibrous capsule around the hematoma, which contains precipitates of calcium that are reported on an imaging as calcified septations^8^ (as in our case). The radiological basis of diagnosis includes ultrasonography, contrast enhanced CT scan, and MRI scan.^14^, ^15^ The USG is useful in determination of the nature of cysts, presence of calcification, septations, and irregularity of the cyst wall. CECT, in addition to this, helps to localize the cyst in exact relations with surrounding organs. However, of all radiological findings, its almost very difficult to distinguish between the true and false cysts and hence the gold standard always remains histopathology.^1^, ^16^ Splenic cysts should be managed via surgical approach if they are symptomatic and larger than 5 cm. The various modalities of treatment in the literature are total or partial splenectomy, percutaneous drainage, cyst aspiration, marsupialization, and partial cystectomy (fenestration, deroofing).^1^, ^7^, ^8^, ^9^ Total splenectomy is reserved for the huge cysts with the cyst wall covered entirely with splenic parenchyma or near hilum or splenic vessels included in the cyst wall.^1^, ^7^, ^8^, ^10^, ^11^ Fenestration is related to poor outcomes and significant recurrence rates.^9^, ^17^ Total splenectomy was the mainstay of treatment in the past, but recent literature favors the concept of splenic preservation, obviously because of the postoperative overwhelming infection.^11^ In our patient, we performed marsupialization and omentoplasty and did not go for the total splenectomy because the cyst wall was not covered with splenic parenchyma, located at the upper pole, and the hilar vessels were not included in the cyst wall.

## 4 CONCLUSION

Splenic pseudocysts are benign entity often secondary to the rupture (traumatic or overlooked trivial trauma) and hematoma resorption. Pre‐operatively its very difficult to differentiate They are usually asymptomatic but symptomatic forms need surgical treatment. Splenic preservation techniques are preferred over a radical approach unless very large entirely spleen covering or hilar cyst.

## AUTHOR CONTRIBUTIONS

**Govinda Prasad Tiwari:** Conceptualization; formal analysis; resources; writing – original draft; writing – review and editing. **Ramchandra Poudel:** Resources; writing – review and editing. **Sabin Nepal:** Resources. **Sushil Dhakal:** Resources.

## FUNDING INFORMATION

None.

## CONFLICT OF INTEREST STATEMENT

Non conflict of interests.

## ETHICAL STATEMENT

This is the retrospective case report and no sampling was used. The ethical approval can be waived.

## CONSENT

Written and informed consent was obtained from the patient for the publication of the case report and is available for the review by the editor of the journal.

## Data Availability

Data described to support the findings in the study are openly accessible in the article.
